# Melatonin Antagonizes Nickel-Induced Aerobic Glycolysis by Blocking ROS-Mediated HIF-1*α*/miR210/ISCU Axis Activation

**DOI:** 10.1155/2020/5406284

**Published:** 2020-05-28

**Authors:** Mindi He, Chao Zhou, Yonghui Lu, Ling Mao, Yu Xi, Xiang Mei, Xue Wang, Lei Zhang, Zhengping Yu, Zhou Zhou

**Affiliations:** ^1^Department of Occupational Health, Army Medical University, 400038 Chongqing, China; ^2^Department of Environmental Medicine, School of Public Health, and Department of Emergency Medicine, First Affiliated Hospital, School of Medicine, Zhejiang University, 310058 Hangzhou, China

## Abstract

Nickel and its compounds, which are well-documented carcinogens, induce the Warburg effect in normal cells by stabilizing hypoxia-inducible factor 1*α* (HIF-1*α*). Melatonin has shown diverse anticancer properties for its reactive oxygen species- (ROS-) scavenging ability. Our aim was to explore how melatonin antagonized a nickel-induced increment in aerobic glycolysis. In the current work, a normal human bronchial epithelium cell line (BEAS-2B) was exposed to a series of nonlethal doses of NiCl_2_, with or without 1 mM melatonin. Melatonin attenuated nickel-enhanced aerobic glycolysis. The inhibition effects on aerobic glycolysis were attributed to the capability of melatonin to suppress the regulatory axis comprising HIF-1*α*, microRNA210 (miR210), and iron-sulfur cluster assembly scaffold protein (ISCU1/2). N-Acetylcysteine (NAC) manifested similar effects as melatonin in scavenging ROS, maintaining prolyl-hydroxylase activity, and mitigating HIF-1*α* transcriptional activity in nickel-exposed cells. Our results indicated that ROS generation contributed to nickel-caused HIF-1*α* stabilization and downstream signal activation. Melatonin could antagonize HIF-1*α*-controlled aerobic glycolysis through ROS scavenging.

## 1. Introduction

Nickel and its compounds are well-known carcinogens for nasal and lung carcinoma in occupationally exposed workers. The International Agency for Research on Cancer (IARC) categorizes nickel (metallic and alloys) as a group 2B carcinogen, while nickel compounds are classified as group 1 carcinogens [1]. Although nickel-associated pulmonary carcinomas have been confirmed by both epidemiologic and experimental evidence, the exact mechanism of nickel carcinogenesis remains unclear. Aerobic glycolysis is considered to play roles in nickel-induced cell transformation [2–4]. On the one hand, the metabolism pattern of tumor cells is characterized by aerobic glycolysis, the so-called Warburg effect [5]. On the other hand, enhanced glycolysis leads to the accumulation of lactic acid in the extracellular space. The reduced pH value is an important feature of the solid tumor microenvironment, which leads to cellular gene and metabolic reprogramming and subsequently prompts tumor progression [6]. Therefore, the process by which normal cells adopt glycolysis is presumed to play roles in the initiation phase of carcinogenesis [7].

The identification of the HIF-1*α*/miR210/ISCU regulatory axis has provided a new explanation for aerobic glycolysis [8]. Under hypoxia conditions, hypoxia-inducible factor 1*α* (HIF-1*α*) escapes from degradation and enhances its transcriptional activity [9]. MicroRNA210, a newly identified target of HIF-1*α*, also has a hypoxia response element for HIF-1*α* binding. Overexpressed miR210 binds to the 3′ untranslated region (UTR) of its target genes and represses their expression. The iron-sulfur cluster (FeS) assembly scaffold protein ISCU is a target of miR210. There are two isoforms of ISCU: ISCU1 is localized in the cytosol and ISCU2 is localized in the mitochondria. Both are repressed by overexpressed miR210. In turn, FeS assembly is disturbed and FeS-dependent enzymes for oxidative phosphorylation (OXPHOS) such as aconitase and NADH-coenzyme Q reductase (complex I) are inactivated, leading to OXPHOS suppression [8]. This axis has been proven to mediate several physiologic and pathologic processes associated with hypoxia [10–13]. Notably, hypoxia is not the only factor that stabilizes HIF-1*α* through prolyl-hydroxylase enzyme (PHD) inhibition. Other factors, such as ROS, succinate, deferoxamine, and CoCl_2_, have been found to cause HIF-1*α* accumulation through different mechanisms [9, 14]. Interestingly, the stabilization of HIF-1*α* by nickel under normoxia is a well-known effect that is believed to play a key role in nickel-associated carcinogenesis [15]. It is quite natural to investigate whether this regulatory axis underlies aerobic glycolysis under nickel exposure conditions.

In recent years, the ability of melatonin to destabilize HIF-1*α* has been repeatedly investigated regarding the anticancer effects of melatonin [16–19]. The vascular endothelial growth factor- (VEGF-) mediated angiogenesis is attenuated by melatonin through destabilizing HIF-1*α* in different tumor cells [20–22]. Melatonin was also reported to inhibit tumor cells through reversing aerobic glycolysis [6, 23]. According to the published literature, melatonin destabilizes HIF-1*α* via diverse routes depending on the cell type or treatment procedure [9]. Sohn et al. reported that miR3195 and miRNA374b account for the melatonin-induced HIF-1*α* mRNA decrease in PC-3 prostate cancer cells [24]. Park et al. reported that melatonin downregulates HIF-1*α* expression through the inhibition of protein translation in prostate cancer cells [25]. Nevertheless, many studies have focused on the protective effect of melatonin on PHD, whose inactivation dramatically causes HIF-1*α* accumulation. In addition to oxygen, ascorbic acid is also necessary for PHD catalytic activity [9]. Melatonin may maintain PHD activity through its antioxidant capability. Considering that multiple metals can stabilize HIF-1*α* [4], investigating whether and how melatonin ameliorates HIF-1*α* accumulation may increase the knowledge of the melatonin protective effect against heavy metal-associated toxicity [26, 27].

In the present study, we investigated the suppressive effect of melatonin on aerobic glycolysis induced by nickel. The mediating roles of the HIF-1*α*/miR210/ISCU1/2 axis were elucidated in a cell model that exposed human bronchial epithelial cells to nonlethal doses of nickel. A pharmacological dose of melatonin (1 mM) in cell culture studies [9] was applied to investigate potential mechanism of melatonin destabilizing HIF-1*α*.

## 2. Materials and Methods

### 2.1. Cell Culture and Treatments

Human bronchial epithelial cells (BEAS-2B) were purchased from the Institute of Biochemistry and Cell Biology (Chinese Academy of Science, Shanghai, China). Cells were cultured in RPMI 1640 medium (HyClone, Logan, UT, USA) supplemented with 10% fetal bovine serum (FBS; HyClone) and 1% *v*/*v* penicillin/streptomycin (Beyotime, Shanghai, China) and were grown at 37°C in a 5% CO_2_ humidified incubator. At the confluence of 75-85%, the cells were subcultured into plates or dishes for treatments. Nickel chloride (NiCl_2_·6H_2_O) and melatonin (Sigma-Aldrich, St. Louis, MO, USA) were dissolved with sterile H_2_O and absolute ethyl alcohol, respectively, and then were diluted to the appropriate concentrations with cells in medium. The doses of NiCl_2_ and melatonin were chosen based on reported studies and our preliminary experiments. At the 18 h post-NiCl_2_ administration, melatonin was added into the wells and incubated with cells for an additional 6 h. Potential HIF-1*α* inhibitors, namely, 2-deoxyglucose (2-DG), *α*-ketoglutarate (*α*-KG), NAC, and rapamycin, were used to treat cells with the same procedure as melatonin. MG132, a proteasomal inhibitor, was applied to treat cells at 10 *μ*M for 1 h.

### 2.2. Cell Viability

The viability of cells is evaluated with a Cell Counting Kit-8 (CCK8, Dojindo, Kumamoto, Japan), which performs colorimetric assays for the determination of the metabolic activity of living cells. Cells were seeded into a 96-well plate (1 × 10^4^ per well) and were exposed to NiCl_2_ for 18 h. Melatonin and bromopyruvic acid (BrPA; 25 *μ*M) were added into the nickel-containing medium which was incubated for an additional 6 h. Next, the medium was replaced with fresh medium containing 10% *v*/*v* CCK8 solution and incubated for 1 h at 37°C, according to the manual of the kit. The optical density (OD) value of each well was determined at a wavelength of 450 nm using a microplate reader (Infinite™ M200, Tecan, Männedorf, Switzerland). Cell viability was expressed as a percent of the control value.

### 2.3. Lactate Dehydrogenase (LDH) Release

LDH release was detected using a Cytotoxicity Detection Kit (Roche, Mannheim, Germany), which measured LDH activity released from the cytosol of damaged cells into the supernatant. Briefly, cells were plated in a 96-well plate (1 × 10^4^ per well) and were maintained in low-serum (1% FBS) medium, which was used to minimize the disturbance of LDH contained in the serum. At the end of treatment, cell-free culture supernatants were collected from each well and were incubated with LDH assay solution at 25°C for 30 min. The OD was measured at 490 nm by subtracting the reference value at 620 nm. Results were expressed as the percentage of maximum LDH release obtained by lysing the cells in 1% Triton X-100.

### 2.4. Glycolysis Assay

Glycolysis level was indicated by the extracellular acidification rate (ECAR) in media surrounding intact cells, which was carried on an extracellular flux analyzer (Seahorse® Bioscience, MA) as described [28]. Cells (1 × 10^4^ per well) were plated into a dedicated microplate (XF96V3 PS; Seahorse Bioscience, MA) and were treated with NiCl_2_ and melatonin. After 10 mM glucose was injected into a well, ECAR was determined by quantifying H^+^-dependent changes in the fluorescence of a proprietary fluorescein complex. The glycolysis level of each group was expressed as a percent of the control value.

### 2.5. PFK Activity Assay

The activity of phosphofructokinase (PFK) was measured using the PFK Activity Colorimetric Assay Kit (Sigma-Aldrich, St. Louis, MO, USA), by which PFK-mediated NADH generation in the reaction mix is reflected by the OD change at 450 nm. Treated cells were homogenized with PFK Assay Buffer. Cell lysis was performed using the Reaction Mix prepared according to the manufacturer's instructions. The OD of the mixtures was measured per 30 s with the microplate reader. A NADH standard was also established for PFK activity calculation. One unit of PFK is the amount of enzyme that generates 1.0 *μ*M of NADH per minute. After normalization of the protein concentration, the PFK activity was expressed as milliunits/mg of protein.

### 2.6. ATP Assay

The adenosine-5′-triphosphate (ATP) content was determined using the ATP Determination Kit (Life Technologies, Carlsbad, CA). The assay is based on luciferase's absolute requirement for ATP in producing light. Cells were seeded into 12-well plates (1 × 10^5^ per well) and were treated with NiCl_2_ and melatonin. After treatment, the cells were lysed with 100 *μ*l of RIPA buffer (Beyotime) and were centrifuged at 10000 × g for 15 min. Next, 20 *μ*l of supernatant was mixed with 100 *μ*l of determination buffer in a white 96-well plate. The luminescence of each well was measured using the microplate reader. ATP standard curves were established, and the ATP content was expressed as nmol/mg of protein.

### 2.7. Lactate Assay

The intracellular lactate content was measured using the Lactate Determination Kit (Jiancheng, Nanjing, China). In this kit, lactate concentration is determined by an enzymatic assay, which results in a colorimetric product. Briefly, 20 *μ*l of cell lysate was reacted with 1 ml of the determination buffer for 10 min at 37°C. The OD of the final mixtures was measured at a wavelength of 570 nm. Standard curves were established, and the lactate concentration was expressed as *μ*M/mg of protein.

### 2.8. Aconitase and Complex I Activity Assays

Colorimetric assay-based typical FeS metabolic enzyme measurements were performed as in a previous study [8]. The activity of aconitase was reflected by the conversion of *cis*-aconitate to isocitrate. In this assay, isocitrate dehydrogenase excess is used, which catalyzes the oxidation of isocitrate into *α*-ketoglutarate. This reaction couples to the reduction of NADP+, which is monitored at 340 nm. The activity of complex I, which is proportional to the rate of NADH oxidation, is measured by a decrease in absorbance at 340 nm. The cells were plated into a 60 mm dish for these two enzyme activity measurements. After appropriate treatment, the cells were collected in Eppendorf tubes, resuspended in hypotonic buffer (120 mM KCl, 20 mM HEPES, 2 mM MgCl_2_, and 1 mM EGTA, pH 7.4), and disrupted by performing ten 1 s pulses using a Vibra-Cell ultrasonic processor (Sonics & Materials Inc., Danbury, CT). The reaction media for aconitase (5 mM sodium citrate, 0.6 mM MnCl_2_, 0.2 mM NADP^+^, 1.5 U isocitrate dehydrogenase, and 50 mM Tris-Cl, pH 7.4) or complex I activity assays (25 mM potassium phosphate, 5 mM MgCl_2_, 0.13 mM NADH, 65 *μ*M ubiquinone 1, 2 mM NaCN, 2 *μ*g/ml antimycin A, and 2.5 mg/ml BSA, pH 7.4) were prepared and prewarmed at 37°C. Next, 20 *μ*l of lysate was mixed with 180 *μ*l of reaction medium in a 96-well plate (*μ*Clear®; Greiner Bio-One, Germany). The activity of aconitase or complex I was indicated by the optical density change of each well at 340 nm and was expressed as a percent of the control value.

### 2.9. Immunofluorescence Staining

For the immunofluorescence assay, cells were grown on gelatin-coated glass coverslips. After appropriate treatments, the cells were fixed with 4% (*w*/*v*) paraformaldehyde in PBS for 30 min, followed by permeabilization with 0.25% Triton X-100 in PBS for 10 min. Next, the cells were blocked with 10% BSA in PBS. The fixed cells were incubated with rabbit anti-ISCU1/2 (1 : 20; Santa Cruz Biotechnology Inc., Santa Cruz, CA) antibody in blocking buffer at 4°C overnight. The slides were then washed five times with PBS and were incubated with an Alexa Fluor® 488 donkey anti-rabbit IgG (H+L) antibody (1 : 500; Invitrogen, Carlsbad, CA) for 1 h at 37°C. DAPI Staining Solution (Beyotime) was used for nuclear counterstaining. The coverslips were mounted on glass slides using Antifade Mounting Medium (Beyotime). The stained samples were examined using a Zeiss confocal laser scanning microscope (Zeiss, LSM 780). The images were captured and processed using Zeiss LSM 780 software.

### 2.10. Western Blotting

Protein samples (20-40 *μ*g) from treated cell extracts were resolved by 10% or 12% SDS-PAGE gel and were transferred to a polyvinylidene fluoride membrane (Bio-Rad, Hercules, CA). The membranes were probed for various proteins using the appropriate antibodies (primary antibody incubated overnight at 4°C, secondary antibody incubated for 1 h at room temperature) and were visualized using an electrochemiluminescence (ECL) system (Thermo Fisher Scientific, Waltham, MA). The bands were imaged and analyzed using the ChemiDoc™ XRS+ System with Image Lab™ Software (Bio-Rad, Hercules, CA). The applied primary antibodies included HIF-1*α* (1 : 1000 Novus Biologicals, Littleton, CO), hydroxy-HIF-1*α* (1 : 1000 Cell Signaling Technology, Danvers, MA), ISCU1/2 (1 : 200 Santa Cruz Biotechnology, Santa Cruz, CA), aconitase 2 (1 : 1000), and NDUFA9 (1 : 2000, Abcam, Cambridge, UK).

### 2.11. Cell Transfection

ISCU1/2 siRNA (h) (sc-270108) and control siRNA (sc-37007) were purchased from Santa Cruz Biotechnology (Santa Cruz, CA). miR210 mimic (miR10000267), mimic control (miR01201), miR210 inhibitor (miR20000267), and inhibitor control (miR02201) were purchased from RiboBio Co. Ltd. (Guangzhou, China). The oligonucleotides were transfected into cells according to the manufacturer's instructions. Briefly, 24 h after cell seeding, medium without penicillin/streptomycin was replaced with the transfection medium containing the oligonucleotides (200 nM ISCU1/2 siRNA, 100 nM miR210 mimic or inhibitor) and 0.2% *v*/*v* Lipofectamine 2000 transfection reagent (Invitrogen, Carlsbad, CA). After 4 h of incubation, the transfection medium was replaced with fresh penicillin/streptomycin-free medium for 24 h before subsequent experiments. HIF-1*α*-specific short hairpin RNAs (shRNA) were designed and packaged into lentivirus by GeneChem Inc. (Shanghai, China). After testing, the most effective construct (target sequence: GCGAAGTAAAGAATCTGAA) was applied in formal experiments. To knock down HIF-1*α*, the cells were seeded into culture plates 1 day prior to infection. The culture medium was replaced by the infection medium containing 5 *μ*g/ml of polybrene and the packed lentivirus with a multiplicity of infection (MOI) of 20. Twelve hours later, the infection medium was replaced with fresh culture medium. Cells were cultured for an additional 48 h before nickel treatment.

### 2.12. qRT-PCR Analysis

The extraction of total RNA and quantification of miR210 were performed following our previously published protocol [3]. The bulge-loop miRNA qRT-PCR primer sets (one reverse transcription primer and a pair of quantitative PCR primers for each set, MQP-0102 and MQP-0201) were designed by RiboBio Co. Ltd. (Guangzhou, China). U6 was utilized as an internal control for miRNA.

### 2.13. Electrophoresis Mobility Shift Assay (EMSA)

Electrophoresis mobility shift assay was applied to test the DNA binding activity of HIF-1*α*. Both biotin-labeled and nonlabeled cold competitor oligonucleotide probes for HIF-1*α* were purchased from Beyotime. The oligonucleotide probe sequences were 5′-TCT GTA CGT GAC CAC ACT CAC CTC-3′ and 3′-AGA CAT GCA CTG GTG TGA GTG GAG-5′. After treatment, cell nuclear proteins were extracted using a nuclear protein extract kit (Beyotime). The reaction mixtures that contained 10 *μ*g of nuclear proteins, 20 nM labeled probes, 2.5% glycerol, 5 mM MgCl_2_, 50 ng/*μ*l poly (dI-dC), 0.05% NP-40 and 1x binding buffer (LightShift™ Chemiluminescent EMSA Kit, Thermo Fisher Scientific) were incubated for 20 min at room temperature. The competition reactions were performed by adding 50-fold excess of unlabeled probe to the reaction mixture, while the negative control reactions had no protein sample. Electrophoresis was performed at 10 V/cm using 6% native polyacrylamide gels, and then the shifted samples were transferred to a positively charged nitrocellulose membrane. The membrane was then UV-cross-linked, blocked, and detected with a gel imaging system (Bio-Rad). Three independent experiments were carried out.

### 2.14. Statistical Analysis

Values are expressed as means ± S.E.M. and were analyzed by SPSS (V18.0.0). Data comparisons were completed using one-way ANOVA or paired *t*-test to compare the means of different treatment groups. *P* < 0.05 was considered statistically significant.

## 3. Results

### 3.1. BrPA Exacerbates the Cytotoxicity of Nickel

**Figure 1:**
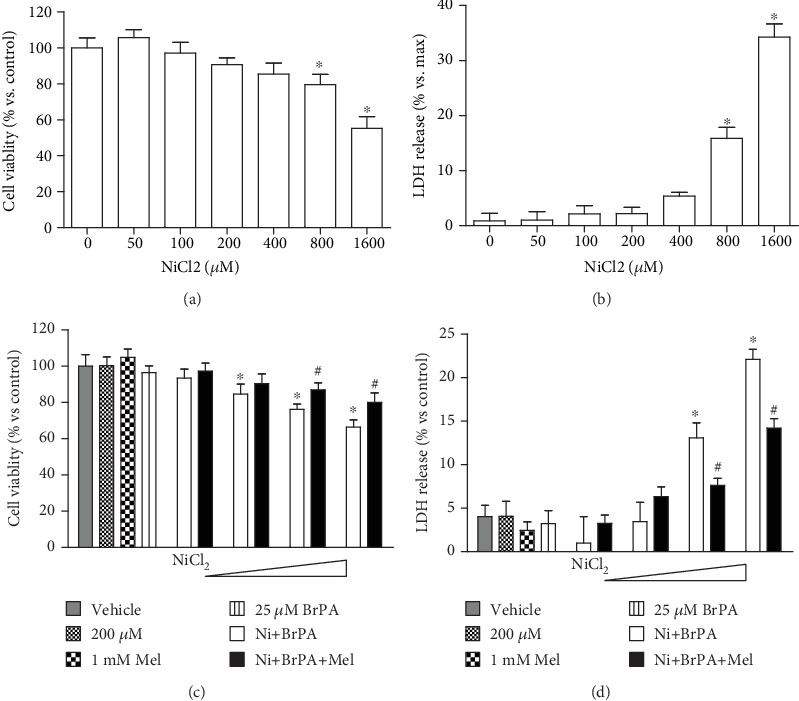
Effect of melatonin on nickel and BrPA coexposure induced cytotoxicity. Single nickel exposure induced cytotoxicity in BEAS-2B cells was measured with cell viability and LDH release experiments. Cells exposed to different concentrations of NiCl_2_ (50-1600 *μ*M) for 24 h (a and b). A glycolysis inhibitor, bromopyruvic acid (BrPA), was applied to coexpose with nickel. At the approach of 18 h post NiCl_2_ administration, 1 mM melatonin was added and incubated with cells for an additional 6 h. The effect of melatonin on BrPA and NiCl_2_ cotreatment induced cell death was measured with cell viability (c) and LDH release (d). The error bar reflects the S.E.M. of at least three independent experiments. ^∗^*P* < 0.05 compared with the nickel-free control group, and ^#^*P* < 0.05 compared with the same nickel concentration but with the melatonin-free group.

Nickel-induced cytotoxicity was measured by cell viability and LDH release assays. Single exposures to NiCl_2_ for 24 h did not induce significant cell death in BEAS-2B cells until the dose was over 800 *μ*M (Figures 1(a) and 1(b)). A single administration of bromopyruvic acid (BrPA), a glycolysis inhibitor, also did not impact cell death. However, the coexposure of NiCl_2_ and BrPA led to cell death, as demonstrated by the cell viability decrease and LDH release increase. These toxicities were ameliorated by melatonin administration (Figures 1(c) and 1(d)). Suppressing glycolysis exacerbates the toxicity of nickel, indicating that aerobic glycolysis may facilitate cell survival under nickel exposure conditions.

### 3.2. Melatonin Antagonizes Nickel-Induced Aerobic Glycolysis

**Figure 2:**
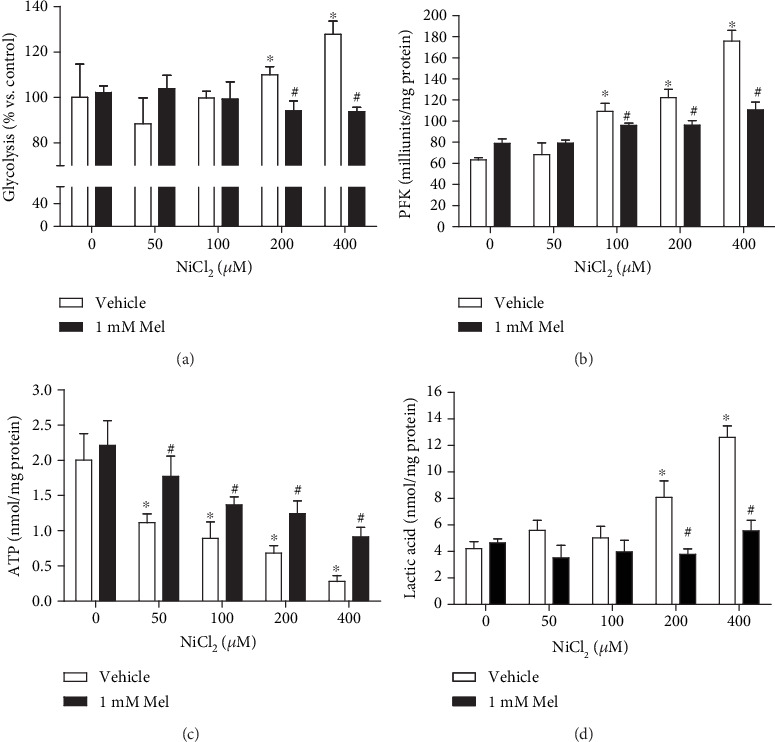
Effects of melatonin on nickel-induced aerobic glycolysis. Cells were exposed to NiCl_2_ (50, 100, 200, and 400 *μ*M) for 24 h. The effects of 1 mM melatonin on aerobic glycolysis in nickel-treated cells were evaluated. The glycolysis levels of cells, indicated by the extracellular acidification rate, were evaluated by a Seahorse® extracellular flux analyzer (a). The activities of phosphofructokinase (PFK), a key enzyme for glycolysis, were measured with a colorimetric based kit (b). Cellular energy metabolite levels, including the concentrations of ATP (c) and lactic acid (d), were measured in treated cells. The error bar reflects the S.E.M. of at least three independent experiments. ^∗^*P* < 0.05 compared with nickel-free control group, and ^#^*P* < 0.05 compared with the same nickel concentration but with the melatonin-free group.

Using a Seahorse® extracellular flux analyzer, the cellular glycolysis levels were manifested by the glucose-induced extracellular proton increase. Glycolysis activity was increased in cells exposed to NiCl_2_ at 200 and 400 *μ*M compared with that in control cells (Figure 2(a)). Phosphofructokinase (PFK) catalyzes fructose-6-phosphate to fructose-1,6-diphosphate, the rate-limiting step of glycolysis. After nickel exposure, the activities of PFK were enhanced in a dose-dependent manner (Figure 2(b)). As a compromised ATP generator, glycolysis shows low efficiency in ATP production. The cellular ATP concentrations were dramatically decreased (Figure 2(c)). Cellular lactic acid, the product of glycolysis, was increased in 200 and 400 *μ*M NiCl_2_-treated cells (Figure 2(d)). Interestingly, melatonin treatment diminished glycolysis levels, PFK activities, and lactic acid increase but restored the ATP levels in nickel-exposed cells (Figures 2(a)–2(d)).

### 3.3. Melatonin Reverses Nickel-Induced Suppression of the Activities of FeS-Dependent Metabolic Enzymes

**Figure 3:**
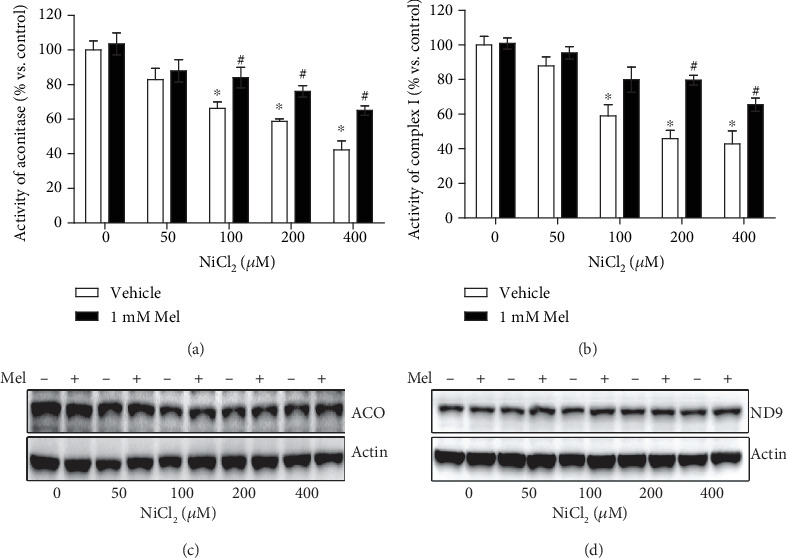
Effects of melatonin on activities of FeS-dependent metabolic enzymes in nickel exposure. The activities of two typical FeS-dependent metabolic enzymes, aconitase and mitochondrial respiratory chain complex I, were measured with colorimetric assays (a and b). The protein levels of these two enzyme components (ACO2 for aconitase, NDUFA9 for complex I) were evaluated with western blot (c and d). ACO: ACO2; ND9: NDUFA9. The error bar reflects the S.E.M. of at least three independent experiments. ^∗^*P* < 0.05 compared with the nickel-free control group, and ^#^*P* < 0.05 compared with the same nickel concentration but with the melatonin-free group.

Cellular OXPHOS depends on a series of FeS-dependent metabolic enzymes, in which FeS acts as a catalysis center. The activities of aconitase and mitochondrial respiratory chain complex I, both of which contain the FeS cluster, were measured after nickel and melatonin treatments. As shown in Figures 3(a) and 3(b), the activities of these two enzymes were suppressed by nickel, but the repression was restored in the presence of melatonin. Notably, the protective effects of melatonin on FeS-containing enzyme activities were not accompanied with the increase of protein amounts of these enzyme components (ACO2 for aconitase, NDUFA9 for complex I), as shown in Figures 3(c) and 3(d). These results indicated the roles of melatonin on maintaining FeS clusters.

### 3.4. Melatonin Restores Nickel-Induced Decrease in the ISCU Protein Levels

**Figure 4:**
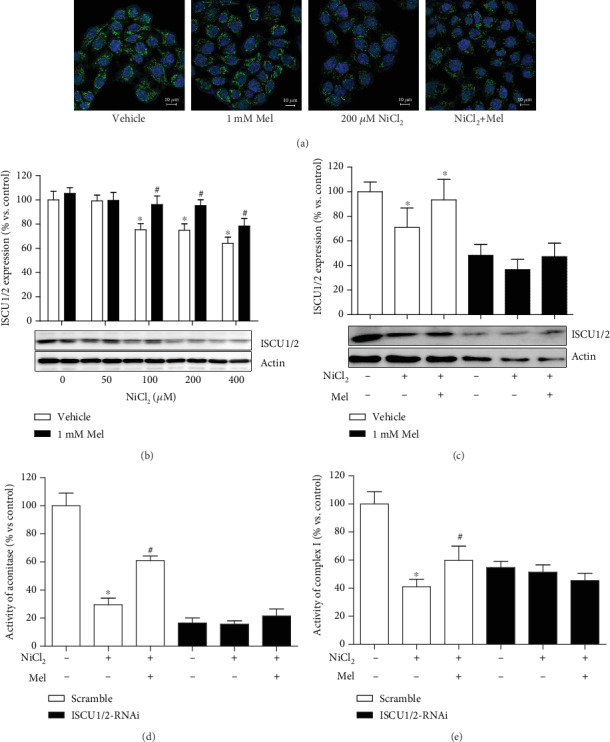
Effects of melatonin on nickel-induced alteration of ISCU1/2 protein levels. The ISCU1/2 protein levels in cells treated with NiCl_2_ and melatonin were evaluated by immunofluorescence staining ((a): green—ISCU1/2; blue—DAPI; scale bar—10 *μ*m) and western blot (b). The expression of ISCU1/2 was silenced by transfection with RNA interference (RNAi) construct. The effects of nickel and melatonin on ISCU1/2 protein levels (c) and the activities of aconitase (d) and complex I (e) were evaluated in transfected cells. The error bar reflects the S.E.M. of at least three independent experiments. ^∗^*P* < 0.05 compared with the nickel-free control group, and ^#^*P* < 0.05 compared with the same nickel concentration but with the melatonin-free group.

To investigate the mechanism of melatonin antagonizing nickel-induced glycolysis, the levels of HIF-1*α*/miR210/ISCU1/2 were analyzed one by one. First, the protein levels of ISCU1/2, the key molecule for FeS assembly, were evaluated by both immunofluorescence staining and immunoblotting. Nickel exposure reduced the protein levels of ISCU1/2. However, these reductions were reversed by melatonin administration (Figures 4(a) and 4(b)). The effects of melatonin that maintained both the protein levels of ISCU1/2 and the activities of FeS-dependent enzymes were diminished by the knockdown of ISCU1/2 expression with ISCU1/2 siRNA (Figures 4(c)–4(e)).

### 3.5. Melatonin Blocks Nickel-Induced Overexpression of miR210

**Figure 5:**
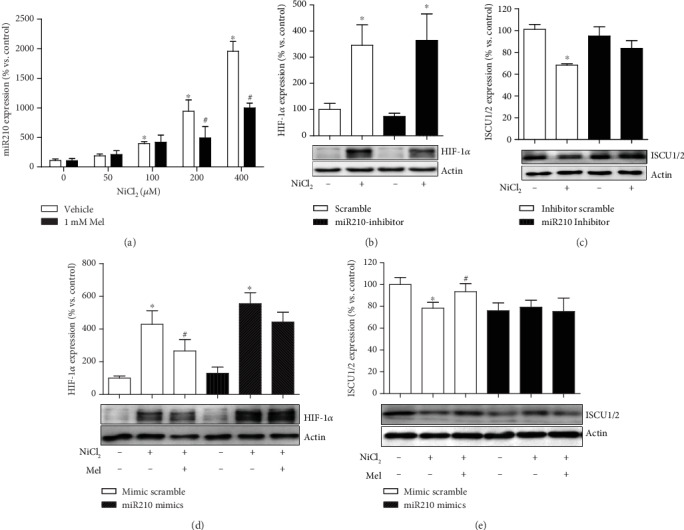
Effects of melatonin on nickel-induced alteration of miR210 expression. The levels of miR210 expression in cells exposed to nickel with or without melatonin were evaluated by qRT-PCR (a). Chemically synthesized oligonucleotides were transfected into cells to control the function of miR210. The effects of melatonin on nickel-caused alternation of HIF-1*α* (b and d) and ISCU1/2 (c and e) were evaluated under conditions of miR210 inhibiting or mimicking. The error bar reflects the S.E.M of at least three independent experiments. ^∗^*P* < 0.05 compared with the nickel-free control group, and ^#^*P* < 0.05 compared with the same nickel concentration but with the melatonin-free group.

In the HIF-1*α*/miR210/ISCU1/2 regulatory axis, ISCU1/2 protein levels are reduced in case of miR210 overexpression. As expected, NiCl_2_ induced a sharp increase in miR210 expression, while melatonin treatment significantly repressed miR210 overexpression (Figure 5(a)). Using chemically synthesized oligonucleotides, we artificially upregulated (miR210 mimics) or downregulated (miR210 inhibitor) the expression of miR210. The miR210 inhibitor did not have an effect on NiCl_2_-induced HIF-1*α* accumulation (Figure 5(b)) but reversed the nickel-induced reduction of ISCU1/2 (Figure 5(c)), similar to melatonin. The miR210 mimics did not have an effect on melatonin mediating HIF-1*α* destabilization (Figure 5(d)) but diminished the effects of melatonin on the ISCU1/2 level (Figure 5(e)).

### 3.6. Melatonin Attenuates Nickel-Induced Accumulation of HIF-1*α*

**Figure 6:**
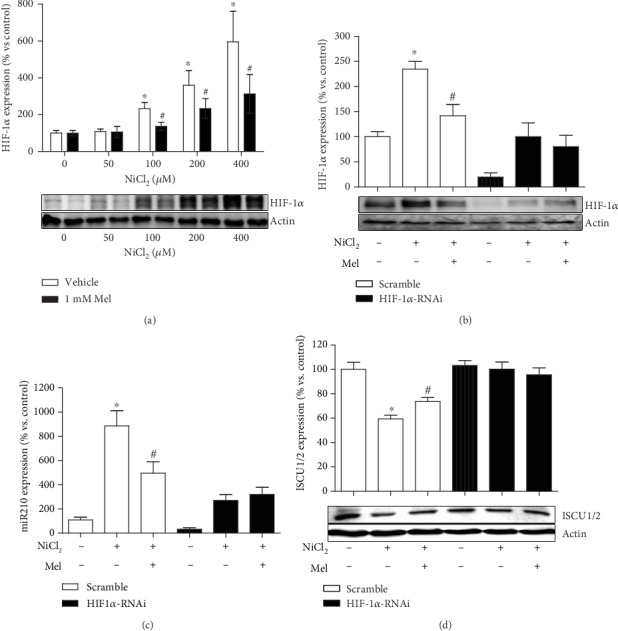
Effects of melatonin on nickel-induced HIF-1*α* accumulation. The levels of HIF-1*α* protein in cells exposed to nickel with or without melatonin were evaluated by western blot (a). HIF-1*α* expression was silenced by transfection with RNAi construct. The effects of melatonin on nickel-caused alternation of HIF-1*α* (b), miR210 (c), and ISCU1/2 (d) were evaluated under condition of HIF-1*α* knockdown. ^∗^*P* < 0.05 compared with the nickel-free control group, and ^#^*P* < 0.05 compared with the same nickel concentration but with the melatonin-free group.

Melatonin remarkably reduced the nickel-induced HIF-1*α* accumulation (Figure 6(a)). Similar to the effect of melatonin, the knockdown of HIF-1*α* expression with lentivirus transfection ameliorated the nickel-induced HIF-1*α* accumulation (Figure 6(b)), miR210 overexpression (Figure 6(c)), and ISCU1/2 protein level reduction (Figure 6(d)). Notably, under the conditions of HIF-1*α* silencing, melatonin treatment did not additionally impact the levels of HIF-1*α*, miR210, or ISCU1/2 (Figures 6(b)–6(d)).

### 3.7. NAC Manifests a Similar Effect with Melatonin on Inactivation of the HIF-1*α*/miR210/ISCU1/2 Axis

**Figure 7:**
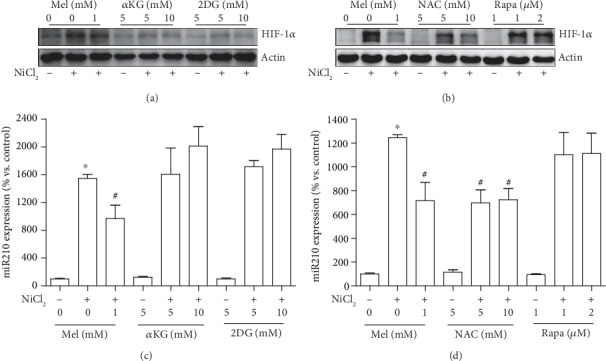
Effects of potential HIF-1*α* inhibitors on nickel-induced HIF-1*α* and miR210 upregulation. Several compounds that reportedly inhibited HIF-1*α* were tested with the indicated concentrations and the same treatment procedure as melatonin. The effects of 2-deoxyglucose (2-DG), *α*-ketoglutarate (*α*-KG) derivative, N-acetylcysteine (NAC), and rapamycin (Rapa) on nickel-induced HIF-1*α* accumulation were measured by western blot (a and b). The effects of HIF-1*α* inhibitors on nickel-induced miR210 increase were measured by qRT-PCR assay (c and d). The error bar reflects the S.E.M. of at least three independent experiments. ^∗^*P* < 0.05 compared with the nickel-free control group, and ^#^*P* < 0.05 compared with the same nickel concentration but with the melatonin- or NAC-free group.

To explore the mechanism by which melatonin ameliorated the nickel-induced HIF-1*α* activation, several compounds that reportedly inhibit HIF-1*α* under specific conditions were tested using the same treatment procedure as melatonin. Two doses of each compound were applied to cover their effect dose range. Among them, 2-DG and the *α*-KG derivative repressed nickel-induced HIF-1*α* accumulation but failed to suppress miR210 overexpression (Figures 7(a) and 7(c)). Rapamycin, an inhibitor of mTOR, had no effects on both HIF-1*α* and miR210 increase. However, N-acetylcysteine, a ROS scavenger, attenuated nickel-induced HIF-1*α* accumulation and miR210 overexpression, similar to melatonin (Figures 7(b) and 7(d)).

### 3.8. Melatonin Preserves PHD Activity through ROS Scavenging

**Figure 8:**
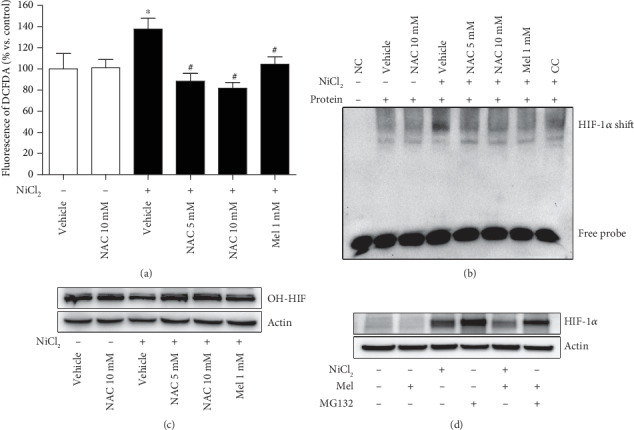
Effect of melatonin and NAC on ROS, HIF-1*α* transcriptional activity, and PHD activity. The effects of melatonin and NAC on nickel-increased cellular ROS level were measured using a DCFDA fluorescence probe (a). The effects of melatonin and NAC on nickel-induced DNA binding activity of HIF-1*α* were evaluated by EMSA assay ((b): NC—no protein control; CC—competition control). The hydroxy-HIF-1*α* (OH-HIF) levels which manifest the activity of prolyl hydroxylases (PHD) were evaluated in treated cells with western blot (c). MG132, a proteasomal inhibitor, also caused HIF-1*α* accumulation. The effects of melatonin to destabilize the HIF-1*α* accumulation induced by nickel or MG132 were evaluated (d). The error bar reflects the S.E.M. of at least three independent experiments. ^∗^*P* < 0.05 compared with the nickel-free control group. ^#^*P* < 0.05 compared with the same nickel concentration but with the melatonin- or NAC-free group.

To further confirm the role of ROS scavenging of melatonin in HIF-1*α* activation, melatonin and NAC were parallel administered in nickel-exposed cells. As expected, both melatonin and NAC ameliorated the nickel-caused ROS level increase (Figure 8(a)). The DNA binding activity of HIF-1*α* triggered by nickel was also suppressed by melatonin and NAC treatment, as shown by EMSA (Figure 8(b)). It has been well established that hypoxia or hypoxia-mimicking chemical agents caused HIF-1*α* accumulation which is mainly attributed to PHD inhibition and subsequent proteasomal degradation repression. Nickel disturbed the activity of PHD, manifesting by a decreased hydroxy-HIF-1*α* level. Both melatonin and NAC abrogated the hydroxy-HIF-1*α* decrease (Figure 8(c)). MG132, a proteasomal inhibitor, also led to HIF-1*α* accumulation for its capability to block protein degradation. Melatonin moderately attenuated MG132-caused HIF-1*α* accumulation, which was less efficient than how it attenuated nickel-induced HIF-1*α* accumulation (Figure 8(d)).

## 4. Discussion

Unlike those well-documented DNA mutagens, such as radiation and benzopyrene, the ability of nickel to break DNA is weak [4, 29]. Although the evidence regarding nickel's relationship with cancer is abundant, the underlying mechanism of nickel carcinogenesis remains unclear. In the present study, we observed that a nonlethal dose of nickel increased glycolysis in BEAS-2B cells. The cell line and nickel dose are comparable to those applied in other nickel carcinogenesis researches [30, 31]. Our finding is in accord with the concept that aerobic glycolysis is a potential contributor for nickel carcinogenesis [2, 32]. Considering that glycolysis is involved in a series of chronic pathological processes, such as carcinogenesis, inflammation, and fibrosis [33–35], the effect of melatonin to block glycolysis may provide a new clue for its pharmacological application [36].

Melatonin is considered a full-service anticancer agent that inhibits cancer initiation, progression, and metastasis [37]. Specific to suppressing aerobic glycolysis, Hevi et al. reported that melatonin mediates energy metabolism through reducing glucose uptake in prostate cancer cells [38]. The administration of melatonin induces Ewing sarcoma cell death. Accompanying cell death, melatonin downregulates glucose uptake, depletes glycogen stores, and attenuates intracellular lactic acid accumulation [23]. More interestingly, physiological nocturnal blood levels of melatonin inhibit the aerobic glycolysis of leiomyosarcoma cells, performed in a model in which tissue-isolated xenografts were perfused in situ with blood collected from healthy adult humans following melatonin supplementation [39]. Compared to previous studies, our study was performed with a normal cell line, rather than a tumor cell line. Our finding may increase the knowledge of melatonin in cancer prevention.

In the present study, we revealed that melatonin suppressed nickel-induced aerobic glycolysis through blocking the HIF-1*α*/miR210/ISCU1/2 axis. This axis has been demonstrated to mediate aerobic glycolysis in multiple physiologic and pathologic processes associated with hypoxia [10–13]. Particularly, in cancer studies, abundant evidence has demonstrated that miR210 is overexpressed in multiple cancers [40]. In the present study, melatonin significantly decreased the level of miR210 through inactivating HIF-1*α*. It is worth further investigating whether miR210 plays a role in melatonin anticancer capacity. Moreover, miR210 repressed the protein level of ISCU1/2, while melatonin maintained the ISCU1/2 level in our study. Considering the importance of FeS-dependent enzymes in energy metabolism, maintaining the ISCU1/2 level may contribute to a well-known protective effect of melatonin that attenuates mitochondria dysfunction [26].

The key step of melatonin suppressing aerobic glycolysis in our present study was to destabilize HIF-1*α*. As reviewed by Vriend and Reiter, melatonin destabilizes HIF-1*α* through different mechanisms, either suppressing synthesis or promoting degradation, according to cell type and treatment condition [9]. Although PHD is considered an oxygen sensor in the cell responses to hypoxia, other factors are also critical for its activity, such as ROS, Fe^2+^, and 2-oxoglutarate. We used several compounds to test the possible mechanism of melatonin destabilization of nickel-induced HIF-1*α*. *α*-KG, the substrate for PHD in HIF-1*α* hydroxylation, blocked nickel-induced HIF-1*α*, while 2-DG, the inhibitor of glucose utilization, had a similar effect. Although *α*-KG and 2-DG destabilized HIF-1*α* in accordance with a previous study [14], both failed to inhibit the upregulation of miR210. A plethora of literature has documented that miR210 is controlled by HIF-1*α* [41]. However, negative feedback by miR210 in inhibiting HIF-1*α* expression was also reported [42]. Whether *α*-KG or 2-DG complicates the cross effects between miR210 and HIF-1*α* warrants further investigation. The mTOR signaling pathway also participates in the transcription and degradation of HIF-1*α* because rapamycin attenuates both the increase in HIF-1*α* protein and transcription activity under low oxygen or CoCl_2_ exposure conditions [43]. In our study, with the same treatment procedure of melatonin, rapamycin did not work out on HIF-1*α* accumulation and miR210 upregulation. We also found that the phosphorylation level of mTOR was suppressed by Ni treatment (data was not shown). These results indicated that the mTOR signaling pathway may play less important roles in nickel-induced HIF-1*α* accumulation.

It has been known for some time that ROS inactivates PHD; however, the significance of ROS in HIF-1*α* signal activation remains controversy [4]. Because ROS are usually also increased in hypoxia, it is hard to clarify which is the primary inducer, lower oxygen or ROS, for PHD inhibition. Regarding the chemical-mimic hypoxia effect induced by cobalt or nickel, a widely accepted hypothesis for PHD inhibition is that they belong to transition metal elements and disturb the homeostasis of the iron ion [44]. However, the disturbance of nickel on iron is not specific to PHD. Because of the unique role of iron in biochemical reactions, any perturbation in iron utilization inevitably induces ROS generation, especially the inactivation of metabolic enzymes in mitochondria [45]. Therefore, the nickel-induced ROS cannot be excluded for HIF-1*α* accumulation. In our present study, both melatonin and NAC abolished the nickel-induced activation of the HIF-1*α*/miR210/ISCU1/2 axis. Based on their well-known ROS-scavenging capability, our data supported that ROS are the main inducers of PHD inhibition in nickel exposure. Additionally, melatonin more efficiently destabilized the nickel-induced HIF-1*α* than it did on MG132-caused HIF-1*α* accumulation. This result indicated that melatonin exerted its effect on PHD, rather than the following proteasomal degradation. However, this result is not in accordance with the study reported by Park et al., in which melatonin significantly reduced MG132-caused HIF-1*α* accumulation [25]. This contradiction may be attributed to the different cell background and MG132 treatment procedure.

**Figure 9:**
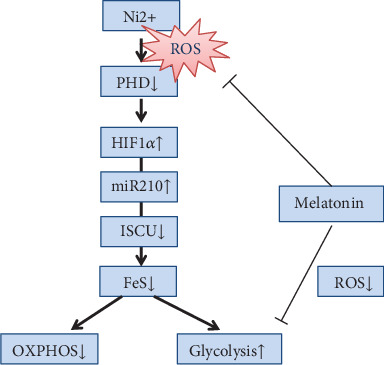
Schematic indicating the role of melatonin in antagonizing nickel-induced aerobic glycolysis. Melatonin prevents the activation of the HIF-1*α*-controlled regulation axis through mitigating the disturbance of ROS on PHD. For more details, see Discussion.

In summary, our study demonstrated that melatonin repressed nickel-induced aerobic glycolysis in normal cells through blocking activation of the HIF-1*α*/miR210/ISCU1/2 axis. This effect depended on its capability of ROS scavenging, by which melatonin maintained the PHD activity in HIF-1*α* degradation (Figure 9). The investigation on ROS-mediated HIF-1*α*/miR210/ISCU axis activation and its roles in aerobic glycolysis may open a new sight into the knowledge of oxidative stress-induced diseases.

## Data Availability

The data used to support the findings of this study are available from the corresponding author upon request.

## Abbreviations

HIF-1*α*: Hypoxia-inducible factor 1*α*
ROS: Reactive oxygen species
miR210: MicroRNA210
ISCU1/2: Iron-sulfur cluster assembly scaffold protein
NAC: N-Acetylcysteine
FeS: Iron-sulfur cluster
OXPHOS: Oxidative phosphorylation
PHD: Prolyl-hydroxylase enzymes
CCK8: Cell counting kit-8
LDH: Lactate dehydrogenase
PFK: Phosphofructokinase
ATP: Adenosine-5′-triphosphate
EMSA: Electrophoresis mobility shift assay
BrPA: Bromopyruvic acid
mTOR: Mechanistic target of rapamycin.
